# Quasi-BIC Modes in All-Dielectric Slotted Nanoantennas for Enhanced Er^3+^ Emission

**DOI:** 10.1021/acsphotonics.2c01703

**Published:** 2023-01-18

**Authors:** Boris Kalinic, Tiziana Cesca, Ionut Gabriel Balasa, Mirko Trevisani, Andrea Jacassi, Stefan A. Maier, Riccardo Sapienza, Giovanni Mattei

**Affiliations:** † Department of Physics and Astronomy, University of Padova, Via Marzolo 8, Padova, I-35131, Italy; ‡ The Blackett Laboratory, Department of Physics, Imperial College London, London, SW7 2BW, United Kingdom; § School of Physics and Astronomy, Monash University, Clayton, Victoria3800, Australia; ∥ The Blackett Laboratory, Department of Physics, Imperial College London, LondonSW7 2BW, United Kingdom

**Keywords:** all-dielectric nanoantenna, quasi-BIC, erbium, decay rate enhancement, nanoslot, metasurface

## Abstract

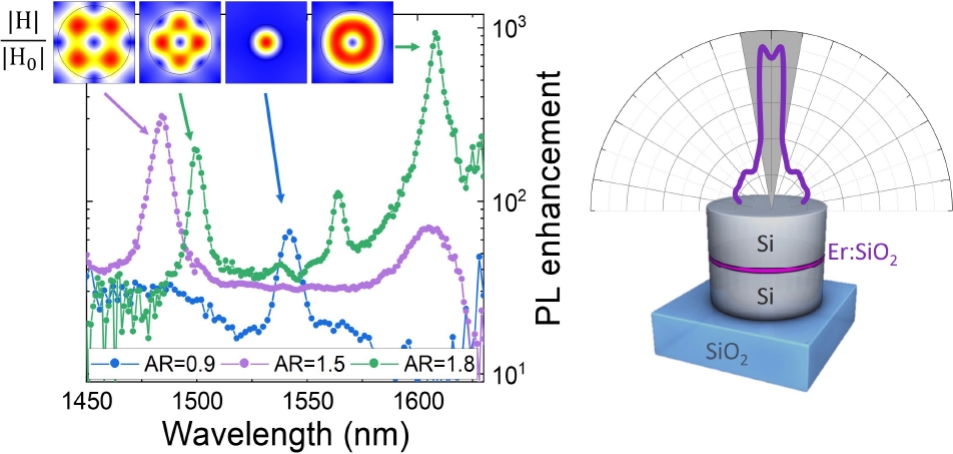

In the quest for new and increasingly efficient photon sources, the engineering of the photonic environment at the subwavelength scale is fundamental for controlling the properties of quantum emitters. A high refractive index particle can be exploited to enhance the optical properties of nearby emitters without decreasing their quantum efficiency, but the relatively modest *Q*-factors (*Q* ∼ 5–10) limit the local density of optical states (LDOS) amplification achievable. On the other hand, ultrahigh *Q*-factors (up to *Q* ∼ 10^9^) have been reported for quasi-BIC modes in all-dielectric nanostructures. In the present work, we demonstrate that the combination of quasi-BIC modes with high spectral confinement and nanogaps with spacial confinement in silicon slotted nanoantennas lead to a significant boosting of the electromagnetic LDOS in the optically active region of the nanoantenna array. We observe an enhancement of up to 3 orders of magnitude in the photoluminescence intensity and 2 orders of magnitude in the decay rate of the Er^3+^ emission at room temperature and telecom wavelengths. Moreover, the nanoantenna directivity is increased, proving that strong beaming effects can be obtained when the emitted radiation couples to the high *Q*-factor modes. Finally, via tuning the nanoanntenna aspect ratio, a selective control of the Er^3+^ electric and magnetic radiative transitions can be obtained, keeping the quantum efficiency almost unitary.

## Introduction

In the weak coupling regime the spontaneous decay rate of an emitter can be modified acting on its electric and magnetic local density of optical states (LDOS), with the magnitude of the radiative emission rate enhancement defined by the Purcell factor (*F*_p_).^1−3^ In recent years, several different designs and configurations of plasmonic nanoantennas and nanocavities have been studied and strong emission enhancements have been obtained.^4−7^ Nevertheless, the presence of a metal nanostructure near the emitter induces also quenching phenomena due to nonradiative energy transfer, thus considerably decreasing the quantum efficiency of the emitting system.^8−11^ This makes plasmonic nanoantennas unsuitable for the realization of high-efficiency photon sources necessary for many cutting-edge applications (e.g., single-photon emitters).^12^ Recently, all-dielectric high refractive index nanostructures have attracted increasing interest due to their unique optical properties (i.e., low absorption, optical magnetism, and multipolar responses),^13−16^ which can be exploited to enhance the optical properties of a nearby emitter without decreasing its quantum efficiency.^17−21^ However, the relatively modest *Q*-factors exhibited by electric and magnetic Mie resonances in nanoparticles such as spheres or cylinders (*Q* ∼ 5–10) have limited the performance of high-index nanostructures in the amplification of the LDOS for resonantly coupled quantum emitters.^22,23^ A possible way to obtain orders of magnitude higher *Q*-factors (up to *Q* ∼ 10^9^) in all-dielectric nanostructures is based on optical bound states in the continuum (BICs).^24−26^ Although true BIC can exist only in structures that are infinitely extended, finite-size systems can support their analog in the form of quasi-BICs.^27,28^ Nanostructures supporting optical quasi-BICs have already demonstrated their ability to outperform traditional photonic nanoparticles for many photophysical processes usually limited either by losses or by low *Q*-factor resonances, such as second- and third-harmonic generation, lasing, light guiding, beam shaping, and sensing.^29−34^ Beside high *Q*, a large Purcell factor requires an ultrasmall mode volume, a concept exploited in many nanoantennas.^35,36^ A nanoslot can do this by exploiting electromagnetic field enhancement at the dielectric discontinuities.^20,37,38^ While combining two resonant antennas usually spoils the collective effect, due to possible interference,^39^ the not-resonant character of nanogaps can be exploited to obtain a synergistic effect.

Here we propose to combine a high Q-factor quasi-BIC with a nanogap. A thin (30 nm) low-index oxide layer doped with quantum emitters (i.e., Er-doped silica) is placed inside high-index slotted silicon nanopillars arranged in a square array. We choose erbium ions in silica as the emitting medium since Er^3+^ can be seen as the ideal candidate for the development of novel photonic quantum sources operating at telecom wavelengths,^40^ due to the sharp room-temperature emission at λ = 1540 nm, that matches the silica minimum absorption window.^41^ We show that the combination of the nanoslot geometry and the quasi-BIC modes can be exploited to boost the electromagnetic LDOS in the optically active region of the nanoantenna. We demonstrate that by coupling the NIR Er^3+^ radiative emission with quasi-BIC resonances supported by silicon slotted nanopillars an enhancement of 2 orders of magnitude of the decay rate and almost 3 orders of magnitude of the photoluminescence intensity can be obtained at room temperature. Furthermore, acting on the nanopillars aspect ratio, a selective control of the Er^3+^ electric and magnetic radiative transitions has been obtained, keeping the quantum efficiency almost unitary. Finally, it was possible also to design and control the emission directivity from the Er:SiO_2_ nanoslot, focusing ∼90% of the total Er^3+^ emitted radiation at λ = 1540 nm in a lobe normal to the sample surface with an angular width of Δθ ∼ 10°.

## Results and Discussion

**Figure 1:**
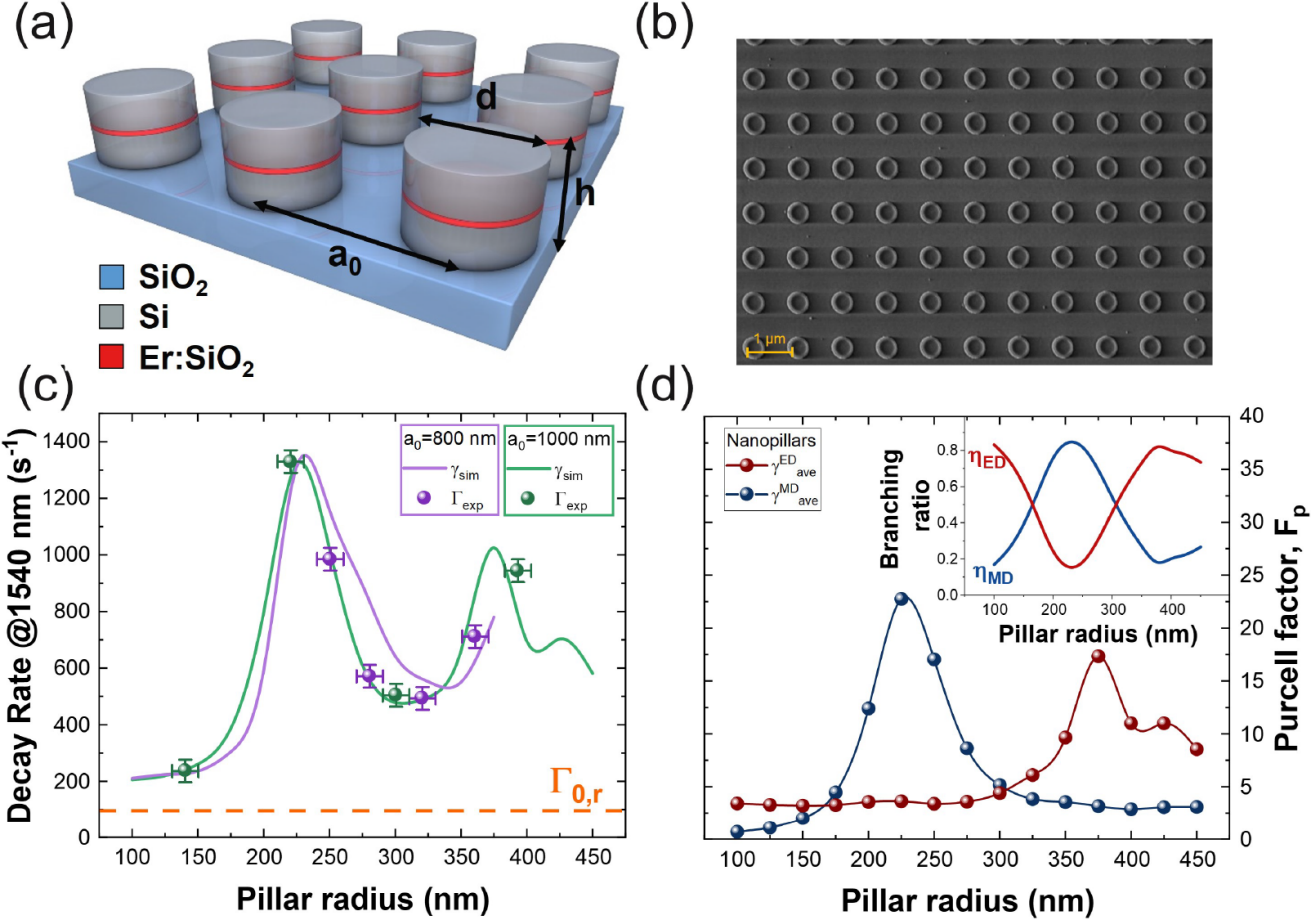
(a) Sketch of the sample structure. (b) SEM image of the sample with *r* = 220 nm and *a*_0_ = 1000 nm (plane view). (c) Simulated radiative decay rate with averaged ED and MD components for an isotropic emitter (continuous lines) and experimentally measured Er^3+^ decay rate (dots) in the confocal setup (NA = 0.85). Green and purple colors indicate the nanopillar array with *a*_0_ = 800 and 1000 nm, respectively. The orange dashed line indicates the Er^3+^ radiative decay rate in bulk silica. (d) Purcell factor in SiO_2_ at λ = 1540 nm for an ED (red) and MD (blue) emitter at the center of the SiO_2_ nanoslot. Inset: electric (η_ED_) and magnetic (η_MD_) dipole branching ratio as a function of the nanopillar diameter.

Figure 1a shows a sketch of the structure of the samples. The emitting layer, a 30 nm thick nanodisk of silica doped with erbium, is placed at half height of the silicon nanopillars arranged in a square array on a silica substrate. The total nanopillar height is *h* = 200 + 30 + 200 = 430 nm. The lattice parameter was set as *a*_0_ = 800 nm and the radius of the nanopillars was varied in the range *r* = 250–350 nm. A second set of samples were fabricated with a larger lattice parameter (*a*_0_ = 1000 nm) and a broader range of pillar radii (i.e., from *r* = 125 to 390 nm), keeping *h* = 430 nm. Thus, the nanopillar aspect ratio (AR = *d*/*h*, being *d* the diameter) was varied from AR = 0.58 to 1.8. An SEM image of the sample with *r* = 220 nm and *a*_0_ = 1000 nm is reported in Figure 1b. The comparison between experimental and simulated transmittance spectra at normal incidence in the 1100–1800 nm wavelength range is reported in Figure S1 of the Supporting Information for the set of samples with *a*_0_ = 1000 nm. The silicon dielectric function used for the simulations was determined by ellipsometry measuring an unpatterned region of the sample. A good agreement between simulated and measured transmittance spectra can be observed.

We investigated the influence of the high-index nanoantenna aspect ratio on the broadband LDOS enhancement in the nanoslot due to the dielectric discontinuities. We demonstrated that more than 1 order of magnitude decay rate enhancement can be obtained for a 30 nm thick slot in the NIR spectral region and that electric and magnetic LDOS exhibit maxima for different silicon nanopillar aspect ratios. Due to the broadband nature of the nanoslot LDOS enhancement, we choose to study the variation of the decay rate at λ = 1540 nm, i.e., the peak wavelength for the ^4^I_13/2_ → ^4^I_15/2_ Er^3+^ transition in silica. The measured Er^3+^ decay rate at λ = 1540 nm as a function of the nanopillar radius is reported in Figure 1c. The purple and green dots indicate samples with a_0_ = 800 nm and *a*_0_ = 1000 nm, respectively. The orange dashed line shows the radiative decay rate of Er^3+^ emitters in a homogeneous SiO_2_ matrix (i.e., Γ_0,*r*_ = 100 s^–1^).^20^ FEM simulated decay rates for a single dipole emitter (λ = 1540 nm) are shown in Figure 1c for the nanopillar arrays with *a*_0_ = 800 nm and *a*_0_ = 1000 nm, purple and green continuous lines, respectively. It is worth underlining that the ^4^I_13/2_ → ^4^I_15/2_ Er^3+^ transition has an almost equal contribution of electric dipole and magnetic dipole components^42^ and therefore the radiative decay rate variation at λ = 1540 nm was calculated by averaging 50–50 the ED and MD decay rates each of which averaged over the 3 spacial directions (i.e., γ_ave_^(ED,MD)^ = 1/3γ_⊥_^(ED,MD)^ + 2/3γ_∥_^(ED,MD)^). To avoid an ambiguous notation, γ is used to indicate the calculated decay rate, while Γ stands for the experimental one. A strong variation of the Er^3+^ decay rate as a function of the slotted nanopillar aspect ratio can be observed. In the explored diameter range, indeed, the measured decay rate varies in the range Γ ∼ 200–1400 s^–1^ exhibiting two distinct maxima at *r* = 220 nm (Γ_exp_ = 1350 ± 50 s^–1^) and at *r* = 380 nm (Γ_exp_ = 950 ± 50 s^–1^), and a local minimum at *r* = 325 nm (Γ_exp_ = 500 ± 30 s^–1^). For nanostructures with *r* < 220 nm the LDOS enhancement in the nanoslot decreases rapidly, reaching the value Γ_exp_ = 210 ± 10 s^–1^ at *r* = 125 nm. The small difference between the decay rates calculated with *a*_0_ = 800 nm and *a*_0_ = 1000 nm (see green and purple curves in Figure 1c) indicates that the lattice parameter has a minor influence on the Purcell factor enhancement at λ = 1540 nm. Besides, nanopillar arrays with a similar aspect ratio (e.g., AR ∼ 1.4) but with a different lattice parameter *a*_0_ exhibit almost equal decay rates, further confirming that the lattice parameter does not play a crucial role in the Er^3+^ LDOS enhancement at λ = 1540 nm for this nanophotonic system. It is worth underlining that a decay rate enhancement of more than an order of magnitude has been measured for all-dielectric nanopillars with *r* = 220 and 390 nm.

To further understand the influence of the aspect ratio of the designed nanoantenna on the electromagnetic LDOS variation in the nanoslot, a set of FEM simulations with electric (ED) and magnetic (MD) dipoles (λ_em_ = 1540 nm) at the center of the SiO_2_ nanoslot have been computed varying the nanopillar radius from *r* = 125 nm to 390 nm, keeping *a*_0_ = 1000 nm. Figure 1d reports the computed Purcell factor (*F*_p_) in the SiO_2_ nanoslot for ED and MD emitters (red and blue curves, respectively) with averaged orientation, i.e., the deconvolution of the green curve in Figure 1c obtained by averaging the electric and magnetic dipole configurations. Figure S2 of the Supporting Information reports the variation of the Purcell factor with respect to the electric and magnetic dipole position inside the silica nanoslot. Although the displacement of the emitter inside the SiO_2_ nanodisk has an influence on the LDOS enhancement, a dipole in the center of the nanoslot with averaged orientation represents a good approximation for the evaluation of the radiative decay rate variation in a SiO_2_ nanoslot homogeneously doped with Er^3+^. The simulated radiative decay rate enhancement for MD emitters presents a maximum at *r* = 220 nm (*F*_p_ = 24). Conversely, electric dipoles exhibit lower Purcell factors with a maximum of *F*_p_ = 17 for nanopillars with *r* = 375 nm. The influence of the number of neighboring nanostructures (finite-size effect) on the decay rate modification has been evaluated by simulating a set of non periodic configurations with a growing number of pillars arranged in a square array with *a*_0_ = 1000 nm around the central one with the emitter (i.e., 1, 9, 25, and 49 pillars in the configuration). The results are reported in Figure S3 of the Supporting Information. The simulated Purcell factor is almost unaffected by the presence of the first, second, and third nearest neighbors square shell in the simulation domain, confirming that the periodic lattice has a small influence on the slotted nanopillar electric and magnetic Purcell factor. The simulated emission system has a unitary quantum efficiency due to the lossless nature of the nanoantenna materials. This has been confirmed by the good agreement between computed and measured values of the decay rate for the whole range of nanopillar radii explored (i.e., green and purple lines vs dots in Figure 1c), indicating that negligible nonradiative decay channels are introduced by the nanoantennas fabrication (i.e., we can assume for Er^3+^ ions a nonradiative rate Γ_nr_ ∼ 0 s^–1^). This point is of paramount importance not only in terms of the PL intensity enhancement but also concerning advanced photonic applications where nonradiative losses are simply not allowed (e.g., single photon sources). The inset of Figure 1d shows the influence of the nanopillar radius on the electric and magnetic branching ratio, defined as the ratio of the electric (magnetic) radiative decay rate and the total decay rate of the emitter (i.e., η_ED_ = γ_ave_^ED^/γ_ave_^tot^, η_MD_ = γ_ave_^MD^/γ_ave_^tot^, where γ_ave_^tot^ = γ_ave_^ED^ + γ_ave_^MD^). A strong modulation of the electric and magnetic dipole contribution to the Er^3+^ radiative transitions can be observed as a function of the nanostructure aspect ratio. A magnetic branching ratio of η_MD_ = 0.85 can be calculated for nanopillars, with *r* = 220 nm and an electric one of η_ED_ = 0.82 for *r* = 375 nm in correspondence to the γ_ave_^MD^ and γ_ave_^ED^ maxima, respectively. Such tunability could be exploited for the selective enhancement of electric or magnetic radiative transitions for emitters with mixed nature such as other rare-earth ions with different ED and MD relative contribution to a specific transition.^43^ The obtained values are much higher than the ones achievable with planar dielectric films with similar slotted structure^20^ and than the ones measured in Reference^44^ for a rare-earth doped thin film deposited on top of a high-index nanopillar array (i.e., γ^MD^/γ^ED^ varies from 0.6 to 1.2). In our nanopillar array, η^MD^/η^ED^ = γ_ave_^MD^/γ_ave_^ED^ ranges from 0.2 to 6.2, demonstrating the superiority of the slot geometry for the effective branching ratio modulation. The strong ED- and MD-selective emission acceleration obtained keeping at the same time the Er^3+^ quantum efficiency close to unity represents an important advantage of the investigated all-dielectric slot nanoantennas with respect to plasmonic nanostructures^45,46^ for applications in quantum photonics which require fast and highly efficient emitters.

**Figure 2:**
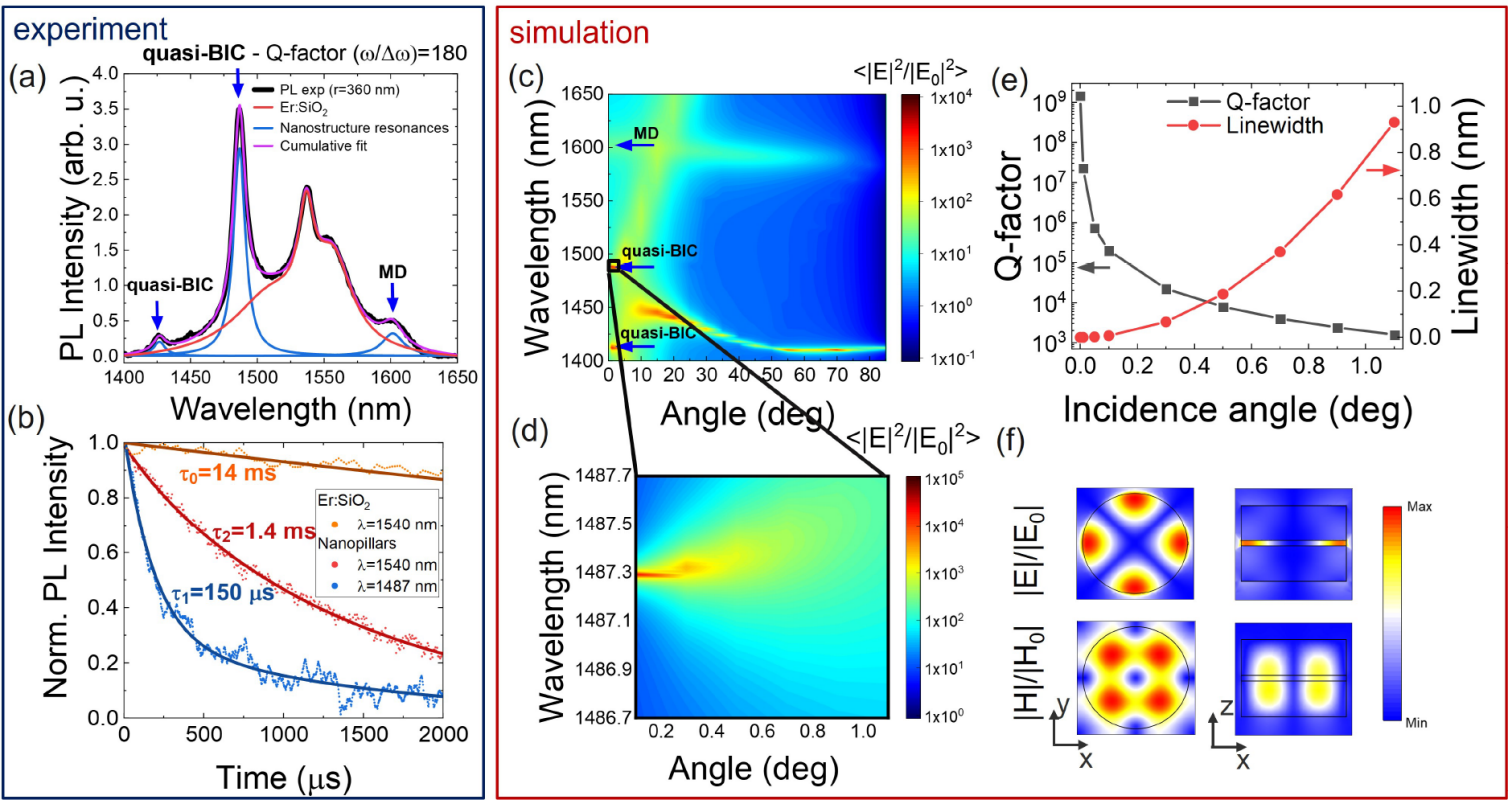
(a) PL emission spectrum of the sample with *r* = 360 nm, *a*_0_ = 800 nm (black line), characteristic PL spectrum of Er^3+^ in SiO_2_ (red line), and peaks associated with the nanopillar modes (blue line). (b) Normalized PL temporal decay for the nanopillar with *r* = 360 nm at λ = 1540 and 1487 nm (red and blue dots, respectively) and for a 30 nm thick Er:SiO_2_ film on the SiO_2_ substrate at λ = 1540 nm (orange dots). The continuous lines are the exponential fits. (c) Wavelength vs angle map of the simulated averaged electric field intensity enhancement in the SiO_2_ nanoslot for a TM-polarized plane wave impinging on the nanopillar array, for the sample with *r* = 360 nm and *a*_0_ = 800 nm. The blue arrows indicate the high *Q*-modes at normal incidence. In (d), a zoom-in view of the region marked with the red square is reported. (e) Simulated *Q*-factor and line width of the quasi-BIC resonance as a function of the incidence angle. (f) Calculated electric and magnetic field enhancement in the nanoslot at λ = 1487 nm at normal incidence.

Even if the increase of the quantum efficiency is mainly controlled by the single nanopillar, the emission directivity and decay rate can be further enhanced by the presence of the nanopillar periodicity. Indeed, ordered arrays of silicon nanopillars arranged in a peculiar resonant configuration can support quadrupolar quasi-BIC modes with huge *Q*-factors (up to *Q* = 10^9^) in the NIR spectral range.^24,26,27^ Thanks to the slotted nanoantenna geometry, in the present work we have exploited quasi-BIC resonances to boost the Er^3+^ radiative emission. Figure 2a shows the PL emission spectrum in the 1400–1650 nm wavelength range for the sample with *r* = 360 nm and *a*_0_ = 800 nm. We have seen that the characteristic Er^3+^ luminescence spectrum in SiO_2_ (indicated by the red continuous line) is altered by the appearance of three additional sharp peaks at λ = 1450, 1487, and 1600 nm (blue lines). By the multiple peak analysis, a *Q*-factor of ∼180 has been calculated for the most intense peak at λ = 1487 nm. This is a clear evidence of the coupling of the Er^3+^ emission with sharp high-*Q* modes supported by the nanoantennas array. The spectral width and the PL intensity of the peak are strongly influenced by the wavelength resolution of the experimental setup (i.e., monochromator slits width) and the numerical aperture of the collection lens. Indeed, the peak intensity and its sharpness are maximized for the smallest collection angle and slits width (see Figure S4(a)), indicating that the measured *Q*-factor can be seen as a lower bound for the true *Q*-factor, which is strongly underestimated due to the finite angular and spectral resolution of the detection setup.

In addition to the LDOS increment due to the high-index slot geometry (as reported in Figure 1c and discussed above), the efficient spectral coupling of the emitters in the nanoslot with quasi-BIC resonances has been exploited to further accelerate the Er^3+^ radiative emission. Figure 2b reports the normalized PL temporal decay at λ = 1540 and 1487 nm for the nanopillar array with *r* = 360 nm and *a*_0_ = 800 nm. The decay curve at λ = 1487 nm is well fitted by a double exponential decay function,  (with *A*_1_ + *A*_2_ = 1), with a dominant short lifetime component with *A*_1_ = 0.90 ± 0.02 and τ_1_ = 150 ± 10 μs and a contribution of a longer component about 10 times smaller (i.e., *A*_2_ = 0.10 ± 0.02 and τ_2_ = 1.40 ± 0.05 ms). Conversely, at λ = 1540 nm the amplitude of the τ_1_ component results almost negligible (i.e., *A*_1_ ∼ 0), and the PL decay is mainly due to the τ_2_ = 1.40 ± 0.05 ms component. This is proof that in correspondence to the quasi-BIC resonance at λ = 1487 nm an order of magnitude stronger decay rate has been obtained (Γ_1_ = 6600 ± 400 s^–1^) with respect to the one measured off-resonance at λ = 1540 nm (Γ_2_ = 710 ± 30 s^–1^). The influence of the numerical aperture of the collection lens and monochromator slits widths on the lifetime estimation is reported in Figure S4(b) of the Supporting Information, pointing out that the contribution of the τ_1_ component at λ = 1487 nm becomes predominant upon the increase of the wavelength and angle resolution of the experimental setup.^47,48^ This provides clear evidence that the huge emission amplification is due to an effective coupling of the Er^3+^ emission with a mode supported by the array with a high quality factor (i.e., quasi-BIC resonance). The orange curve in Figure 2b indicates the decay rate of a reference sample with a 30 nm thick Er:SiO_2_ film on the silica substrate, fitted with a single exponential curve with τ_0_ = 14.0 ± 0.5 ms (Γ_0_ = 70 ± 3 s^–1^). The measured decay rate is in good agreement with the values reported in the literature for Er-doped thin films on the SiO_2_ substrate,^41,49^ proving the high quantum efficiency of the deposited Er:SiO_2_ layer. Moreover, the good agreement between experiments and FEM simulations at λ = 1540 nm for the whole range of nanopillar radii and lattice parameters explored (see Figure 1c) demonstrates that the almost unitary quantum efficiency of the Er:SiO_2_ layer is preserved also after the patterning step necessary for the nanoantenna fabrication. Decay rate measurements have clearly demonstrated that the proposed nanoantenna design can support a strong light–matter interaction inside the SiO_2_ slot, especially when the emitter couples with quasi-BIC modes. At the resonance condition, indeed, a Purcell factor of 2 orders of magnitude has been measured at room temperature (i.e., Γ_1_/Γ_0_ = 94) for a lossless nanostructure (i.e., Γ_nr_ ∼ 0 s^–1^ and Q.E. ∼ 1).

Due the inherent difficulties in simulating an isolated emitter in a periodic structure, we exploited the optical reciprocity principle allowing us to take into account the periodicity of the structure and the spatial distribution of the emitters in the active layer (i.e., the Er:SiO_2_ slot).^44^ According to the reciprocity principle the electric or magnetic LDOS (ρ_e_, ρ_m_) is proportional to corresponding field enhancement at the emitter position for a plane wave impinging on the nanostructure (i.e., ρ_e_ ∝ ⟨|**E**|^2^/|**E**_0_|^2^⟩ and ρ_m_ ∝ ⟨|**H**|^2^/|**H**_0_|^2^⟩, respectively). Figure 2c reports the simulated average electric field intensity enhancement in the SiO_2_ slot  for a TM-polarized plane wave impinging on the nanopillar array. **E** is the simulated local electric field as a function of the incidence polar angle θ (the azimuthal angle was kept ϕ = 0), **E**_0_ is the incident electric field and ⟨ · ⟩ denotes the volume average inside the SiO_2_ slot. For completeness, Figure S5 shows the electric and magnetic field enhancement map for a TE- and TM-polarized plane waves impinging on the nanopillar array along the Γ–X direction in the first Brillouin zone of the reciprocal lattice. In the 1400–1650 nm wavelength range, when the incidence angle approaches the direction normal to the sample surface, three resonances appear in the simulated spectrum (marked with blue arrows in Figure 2c), where the electromagnetic field results strongly amplified. The spectral position of these resonances corresponds to the sharp peaks observed in the measured PL spectrum. The zoom-in view of the mode peaked at λ ∼ 1487 nm (Figure 2d) shows that a giant field intensity enhancement (), with an extremely narrow spectral width (Δλ < 10 pm), can be obtained at an incidence angle approaching the quasi-BIC condition (e.g., at θ = 0.1°) in the nanopillar region corresponding to the emitting layer. Figure S6 of the Supporting Information shows the simulated electric field enhancement in the nanoslot and the far-field reflectance spectra for a plane wave impinging at θ = 0.1° on the structure. The reflectance has the asymmetric Fano-like line-shape, with its asymmetry parameter *q* → 0 at resonance. In addition, Figure 2e shows that, as the incident radiation approaches the resonant condition (i.e., θ = 0°), the line width of the mode becomes vanishingly small, and accordingly its *Q*-factor increases enormously (up to *Q* ∼ 1.5 × 10^9^, a value limited by the numerical precision of the simulation). These features are distinctive of quasi-BIC modes supported by arrays of all-dielectric nanoresonators.^24,26^Figure S7 of the Supporting Information reports the influence of the lattice parameter on the resonance *Q*-factor for the sample with *r* = 360 nm. The highest *Q*-factor has been calculated for the configuration with the densest array of nanopillars (i.e., *a*_0_ = 800 nm). From the electric and magnetic near-field enhancement at λ = 1487 nm shown in Figure 2f, it can be noticed that the resonance has a quadrupole-like field distribution, exhibiting four lobes where the electric or magnetic field results strongly increased. Figure S8 of the Supporting Information shows *y*- and *z*-components of the electric and magnetic fields and the vector field map at λ = 1487 nm.

As a reference, a similar array of Si nanopillars without the SiO_2_ slot was also simulated and a similar quadrupole quasi-BIC resonance (with *Q* ∼ 10^9^) was found at a slightly longer wavelength (see Figure S9 in the Supporting Information). The *x*–*z* view in Figure S9 shows that the low index material of the slot (SiO_2_) increases the electric field enhancement in the emitting layer by roughly 3 times. Therefore, slotted Si nanopillars can be considered a significant advancement with respect to all-silicon nanopillars without the slot, since they preserve the activation of quasi-BIC modes with high *Q*-factors and, at the same time, they are able to tailor the emitter position in the region with the highest LDOS enhancement.

**Figure 3:**
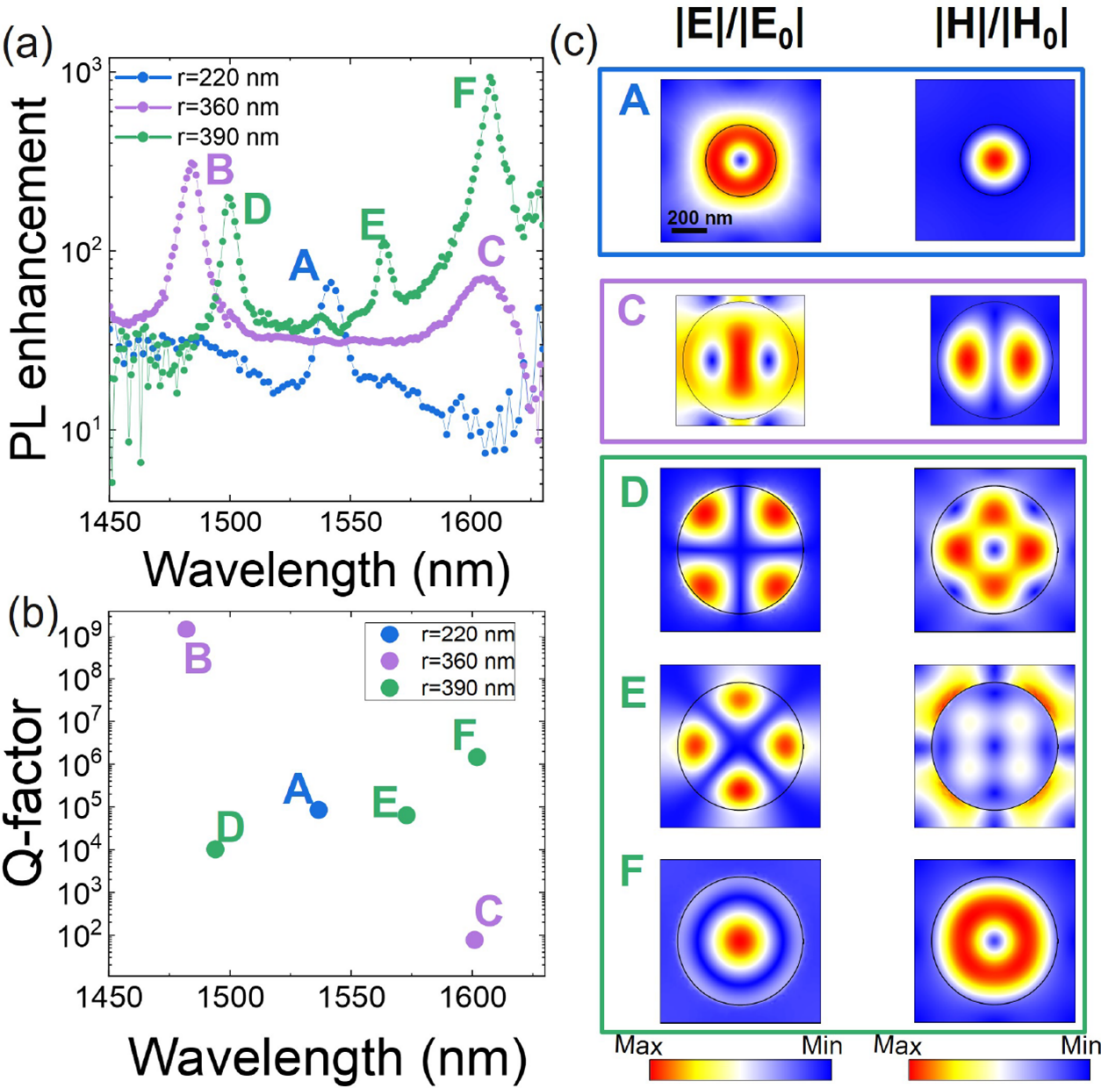
(a) Measured Er^3+^ PL intensity enhancement in logarithmic scale in the 1450–1630 nm wavelength range with respect to a 30 nm thick planar nanoslot. (b) *Q*-factor for the eigenmodes computed by the modal analysis. (c) Electric and magnetic field distribution in the SiO_2_ slot for the modes calculated by the modal analysis. The electromagnetic field distribution of the B resonance is shown in Figure 2e.

Figure 3a shows the measured Er^3+^ PL intensity enhancement with respect to a 30 nm thick planar Si slot for a selected set of samples with quasi-BIC resonances in the Er^3+^ wavelength emission range. The PL signal was collected normal to the surface of the sample with NA = 0.05. A PL enhancement of more than 1 order of magnitude was measured over the whole Er^3+^ emission spectrum. This confirms that the periodic nanopillar geometry strongly favors the out-coupling of the emitted radiation toward the far-field with respect to the planar film structure, as discussed in detail in ref (20). Furthermore, in correspondence to quasi-BIC resonances (labeled with capital letters from A to F in Figure 3) the Er^3+^ PL intensity is increased up to 3 orders of magnitude with respect to the planar configuration. This clearly shows that the photon emission from the nanoslot is strongly amplified when the coupling with quasi-BIC resonances is achieved. In the explored wavelength range, six eigenmodes with extremely high *Q*-factors (up to *Q* ∼ 10^9^) have been found. The resonances with *Q* > 10^2^ are reported in Figure 3b. The huge *Q*-factor exhibited by the eigenmodes computed in the slot nanoantennas is consistent with the excitation of optical quasi-BICs.^15,24,26^ The spectral position of the eigenmodes is in good agreement with the sharp peaks measured in the PL spectra. Considering the electric and magnetic field configuration at the resonances (see Figure 3c), it emerges that the resonance labeled with A and F are toroidal dipole (TD) BIC eigenmodes with dominant ED and MD moment,^24,50^ respectively, while B, D, and E present the characteristic quadrupole-like electromagnetic near-field distribution. The C mode is a broader Mie-type magnetic dipole resonance^15^ with a relatively modest *Q*-factor (*Q* < 10^2^) and correspondingly a lower PL enhancement is obtained (see Figure 3a).

It is worth saying that a *Q*-factor of *Q* ∼ 10^6^ in the 1450–1650 nm wavelength range corresponds to an ultranarrow resonance line width Δλ ∼ 10^–3^ nm, which is extremely challenging to be measured from the experimental point of view. Moreover, quasi-BIC resonances are extremely narrow also from an angular point of view considering the emission directivity (e.g., see Figure 2e). Hence, we expect that the monochromator resolution (Δλ_min_ ∼ 2 nm), the numerical aperture of the collection lens (NA = 0.05) largely contribute to broaden the width of the measured resonances.^47^ In addition, the average random emitter orientation in the SiO_2_ nanoslot decreases the number of erbium ions able to efficiently couple with these highly directional modes and reduces the measured intensity of the resonance. Finally, since mathematical quasi-BIC consist of infinite arrays of identical nanostructures, the finite size effect of the periodic arrays^51^ and nanofabrication imperfections^52^ can decrease significantly the measured *Q*-factor. Since the fabricated nanopillar arrays are 400 × 400 μm large (i.e., containing more than 10^5^ nanoantennas), a marginal effect is expected in the *Q*-factor of the quasi-BIC modes due to the finite size of the structure.^48,51^Figure S10 of the Supporting Information shows the effect of a distribution of nanoparticles radii on the resonance *Q*-factor for the sample with *r* = 360 nm and *a*_0_ = 800 nm, suggesting that nanofabrication imperfections can contribute to limit the maximum *Q*-factor achievable experimentally. Despite all these limiting effects, it is worth highlighting that we have measured a boost of the Er^3+^ photon emission (at telecom wavelength) by almost 3 orders of magnitude at room temperature in a relatively simple photonic geometry that does not need too complex nanofabrication processes.

**Figure 4:**
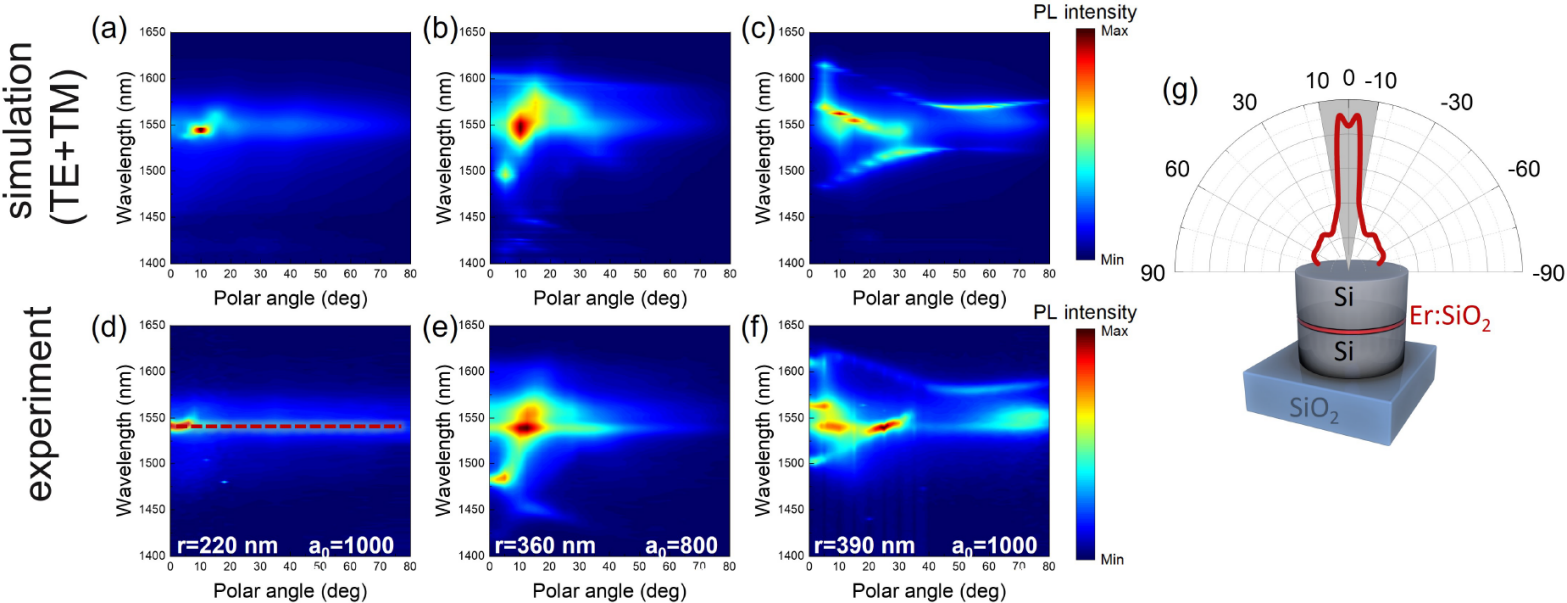
Emission directivity. PL intensity distribution calculated by eq 1 for slotted nanopillars with (a) *r* = 220, (b) 360, and (c) 390 nm. (d–f) Measured angular distribution in the 1400–1650 nm wavelength range for the corresponding samples. (g) Measured angular distribution of the Er^3+^ PL intensity at λ = 1540 nm for the sample with *r* = 220 nm and *a*_0_ = 1000 nm.

Finally, we have designed the nanoantennas array emission directivity. We have exploited the coupling of Er^3+^ emission with quasi-BIC modes to tailor the angular distribution of the PL emission, obtaining a strong beaming effect. To calculate the angular distribution of the Er^3+^ PL emission, the approach based on the reciprocity principle has been adopted. Since the Er^3+^ PL signal was collected along the Γ–X direction of the first Brillouin zone in the reciprocal lattice, keeping constant the azimuthal angle (ϕ = 0 in the present case), the PL intensity as a function of the emission wavelength and the polar angle (θ) can be estimated as^53−55^
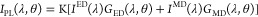
1with:
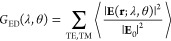

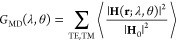
where K is a constant that accounts for the collection efficiency of the experimental setup, **E**(**r**; λ, θ) and **H**(**r**; λ, θ) are the FEM simulated electric and magnetic local field for TE- and TM-polarized plane waves impinging on the nanostructure from the air half-space, ⟨ · ⟩ denotes the volume average inside the emitting layer (i.e., the Er:SiO_2_ slot), and I^ED^(λ) and I^MD^(λ) are the Er^3+^ PL emission intensities in SiO_2_ for the limiting cases of η_ED_ = 1 and η_MD_ = 1, respectively. Using eq 1, the PL intensity distribution maps have been estimated and are reported in Figure 4a–c for the set of samples with *r* = 220, 360, and 390 nm. Figure 4d–f show the corresponding measured angular distribution of the Er^3+^ PL intensity in the 1400–1650 nm wavelength range. The line-shape of the Er^3+^ PL intensity in the 1400–1650 nm wavelength range is significantly altered by the branching ratio. Figure S11 shows the limiting cases for η_ED_ = 1 and η_MD_ = 1, calculated from a set of samples with the Er:SiO_2_ emitting layer placed at different distances from a gold mirror.^42,45^ The PL maps present maxima of the emission intensity in correspondence to specific (θ, λ) configurations, where the emitted light couples to modes supported by the nanopillar array. For example, it is worth noticing that the sample with *r* = 220 nm and *a*_0_ = 1000 nm shows a PL maximum at λ = 1540 nm for a collection angle close to the normal to the sample surface (see Figure 4g), and about 90% of the emitted radiation is focused in a Δθ ∼ ±10° lobe normal to the sample surface where the Er^3+^ PL couples with the toroidal dipole BIC resonance (i.e., resonance labeled with A in Figure 3). This proves that not only decay rate enhancements, but also strong beaming effects can be achieved when the coupling of Er^3+^ luminescence with high-*Q* modes is carefully designed. Our findings demonstrate that slotted nanopillars are highly promising candidates for the development of more efficient or novel light sources. For example, by decreasing the emitter concentration by roughly 2 orders of magnitude, bright, lossless, and highly directional single-photon sources operating at telecom wavelength could be realized.

## Conclusion

We have designed and realized all-dielectric lossless nanoantennas able to boost the Er^3+^ photon emission in the telecom wavelength band and at room temperature. We have shown that ultrahigh *Q*-factor resonances (*Q* > 10^5^) are supported by square arrays of silicon nanopillars with a 30 nm thick Erbium-doped SiO_2_ slot placed at half of the nanopillar height. The eigenvalue analysis performed by FEM simulations evidenced electric and magnetic dipole and quadrupole quasi-BIC modes. These modes are characterized by a giant local density of optical states enhancement inside the nanopillars and specifically in the region corresponding to the optically active layer. Two orders of magnitude decay rate enhancement and almost 3 orders of magnitude PL intensity increment have been measured at room temperature for the Er^3+^ radiative emission in the range λ = 1400–1650 nm when the coupling with quasi-BIC resonances is achieved. Furthermore, we have demonstrated that the slot geometry can be exploited to obtain selective emission enhancements for Er^3+^ electric and magnetic radiative transitions at λ = 1540 nm and that by tailoring the nanopillar aspect ratio from AR ∼ 0.5 to AR ∼ 2 the emitter magnetic branching ratio can be varied from η_MD_ = 0.18 to 0.85, keeping the quantum efficiency almost unitary. Finally, by computing the angularly resolved electromagnetic field enhancement inside the nanoslot it was possible to predict and control the emission directivity of the nanoantenna, focusing more than 90% of the emitted radiation in a lobe normal to the sample surface when the coupling with quasi-BIC resonances is achieved.

## Methods

### Sample Preparation

A sketch of the sample structure is shown in Figure 1a. To realize the slotted nanopillars (Figure 1a), as the first step silicon thin films (200 ± 10 nm thick) were deposited by magnetron sputtering on a silica slab (Herasil, by Heraeus). A 2″ diameter Si target was mounted on a DC source and the deposition was performed at a power *P* = 100 W and a pressure *p* = 5 × 10^–3^ mbar. Before the deposition, the silica substrates were cleaned in a “piranha” solution (3:1, H_2_SO_4_/H_2_O_2_) for 1 h and rinsed in ultrapure water. Erbium-doped silica (Er:SiO_2_) thin films (30 ± 1 nm thick) were also deposited by means of magnetron cosputtering. A metallic erbium target (diameter 2″, thickness 1/4″, purity 99.99%, by K. J. Lesker) was mounted on the DC source, while a silicon dioxide target (diameter 2″, thickness 1/8″, purity 99.9%, by K. J. Lesker) was placed on a radiofrequency (RF) source. The depositions were performed in a slightly oxidizing atmosphere (95% Ar + 5% O_2_) in order to obtain stoichiometric SiO_2_ layers.^56^ Both sources were tilted of about 25° to aim at the center of the sample holder, which was kept under rotation to ensure homogeneity of the deposited films. The power of the two sources was set to keep the Er concentration at 0.3 at. % below the concentration quenching limit ([Er]<1 at. %). Rutherford backscattering spectrometry (RBS) was used to confirm the Er concentration and to exclude the presence of contaminants in the Er:SiO_2_ layer. Thickness and roughness of the deposited films were determined by atomic force microscopy (AFM) measurements with a NT-MDT Solver-Pro AFM in noncontact mode. The dielectric functions were determined by means of a Woollam variable angle spectroscopic ellipsometer (VASE).

After the deposition of Si/Er:SiO_2_/Si multilayers on silica substrates, the samples were further cleaned in a “piranha” solution and annealed at 850 °C for 2 h in vacuum (*p* ∼ 5 × 10^–5^ mbar) to promote silicon densification and crystallization and to activate the Er^3+^ luminescence.

Then, an area of 400 × 400 μm^2^ was patterned with a square array of nanopillars by means of electron beam lithography (EBL) and reactive ion etching (RIE). Two sets of samples with square lattice parameters *a*_0_ = 800 and 1000 nm were fabricated. The pillar radius was varied from *r* = 125 to 390 nm, while the pillar height (*h*) was kept at *h* = 430 ± 10 nm.

### FEM Simulations

FEM simulations were carried out in the frequency domain using the commercial software COMSOL Multiphysics and with EMUstack.^57^ Due to the difficulties associated with the simulation of a single emitter in periodic structures, two different computational approaches were exploited. To calculate the radiative decay rate variation at λ_em_ = 1540 nm, the Er^3+^ emitters were modeled as electric or magnetic dipoles,^43^ oscillating at the frequency corresponding to their emission. Each configuration was modeled with a single emitter placed at a specific position in the simulation domain and with a given orientation. To evaluate the finite-size effect on the Purcell factor, i.e., the influence of the neighboring nanostructures on the decay rate modification, configurations with 1, 9, 25, and 49 nanopillars in the simulation domain were considered (as discussed in the Supporting Information). The dipoles were oriented along the *x*-, *y*-, and *z*-axis. The isotropic orientation was calculated averaging with equal weight over the three orientations. To compare with the experimental measurements the electric and magnetic dipole results were averaged with a 50:50 weight, considered the 50:50 nature of the Er^3+^ emission at 1540 nm.^58^ Moreover, the approach based on the optical reciprocity principle^45,55^ was used to study the Er^3+^ emitters coupling with modes that require periodic structures (e.g., quasi-BICs). The modal analysis (computed by the eigenfrequency solver implemented in the commercial software COMSOL Multiphysics) and the electromagnetic field enhancement calculations in the slot were carried out with a square unit cell containing a single slotted nanopillar and applying periodic boundary conditions in the *x*–*z* and *y*–*z* planes and perfectly matching layers along the *z*-direction.

### PL Measurements

PL measurements were performed using as the excitation source a cw fiber-coupled laser diode (λ_ex_ = 520 nm, *P* = 50 mW) mechanically chopped at *f* = 20 Hz. Two different optical set-ups were used to collect the Er^3+^ radiative emission corresponding to the ^4^I_13/2_ →^4^I_15/2_ transition. Time-resolved photoluminescence measurements at λ = 1540 nm (Figure 1b) were taken in a confocal configuration using an objective with numerical aperture NA = 0.85 to maximize the signal-to-noise ratio and the acceptance angle. For the angle resolved PL measurements, the laser diode was mounted on a rotating stage, allowing for the collection of the emitted radiation from θ = 0° to ±90° without altering the angle of incidence between the pumping beam and the sample surface (θ_inc_ = 30°). The PL emission was collected with a converging lens (focal length *f* = 10 cm). An iris was used to vary the numerical aperture of the collecting system in the range NA = 0.05–0.2. The collected PL signal was focused on the entrance slit of a single grating monochromator coupled with a N_2_-cooled photomultiplier tube (HAMAMATSU R5509-72).

## Supporting Information Available

Simulated and measured transmittance spectra at normal incidence, measured Si dielectric function, the variation of the Purcell factor with respect to the electric and magnetic dipole position inside the nanoslot, the radiative decay rate modification as a function of the number of nanopillars in the simulated domain, field intensity maps in the SiO_2_ slot, the influence of the numerical aperture of the collection lens and of the monochromator slits width on the PL spectrum, simulated field intensity and reflectance spectra for the sample with *r* = 360 nm and *a*_0_ = 800 nm, the influence of the lattice parameter on the *Q*-factor, near-field maps for the resonance with *Q* ∼ 10^9^, the influence of nanofabrication imperfections on the *Q*-factor, the field distribution in the nanopillar with and without the slot, and Er^3+^ ED and MD PL intensity spectra (PDF)
