# Supplementary material for: Quasi-BIC Modes in All-Dielectric Slotted Nanoantennas for Enhanced Er3+ Emission
Source: ACS Photonics. 2023 Jan 18;10(2):534–43. doi: 10.1021/acsphotonics.2c01703 (PMC9936627; doi:10.1021/acsphotonics.2c01703)
Supplement: Supplementary file 1 — ph2c01703_si_001.pdf [file ph2c01703_si_001.pdf]

Quasi-BIC modes in all-dielectric slotted nanoantennas for enhanced Er^{3+} emission

Boris Kalinic,[*,†] Tiziana Cesca,[†] Ionut Gabriel Balasa,[†] Mirko Trevisani,[†] Andrea Jacassi,[‡] Stefan A. Maier,[¶,§] Riccardo Sapienza,[‡] and Giovanni Mattei[†]

†*Department of Physics and Astronomy, University of Padova, Via Marzolo 8, Padova, I-35131, Italy*

‡*The Blackett Laboratory, Department of Physics, Imperial College London, London, SW7 2BW, United Kingdom*

¶*School of Physics and Astronomy, Monash University, Clayton, Victoria, 3800, Australia*

§*The Blackett Laboratory, Department of Physics, Imperial College London, London SW7 2BW, United Kingdom*

E-mail: boris.kalinic@unipd.it

SUPPORTING INFORMATION

Transmittance spectra and silicon refractive index

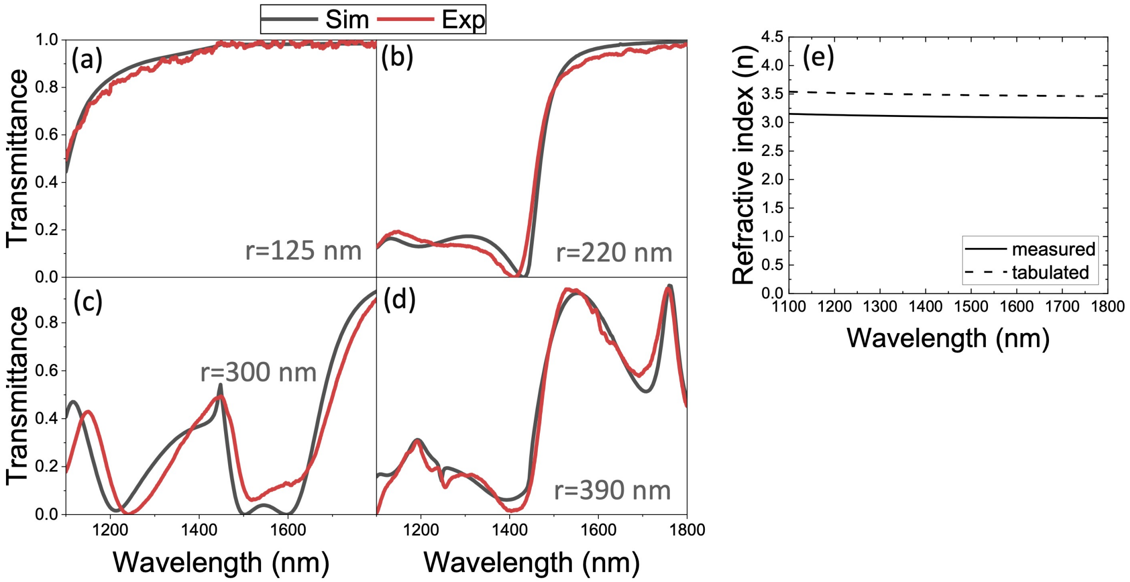

Figure S1: (a-d) FEM simulated (black line) and measured (red line) transmittance spectra at normal incidence for the samples with lattice parameter a_0=1000 nm. (e) Tabulated[1] and measured values of the silicon refractive index (dashed and continuous lines, respectively)

Dipole position in the nanoslot

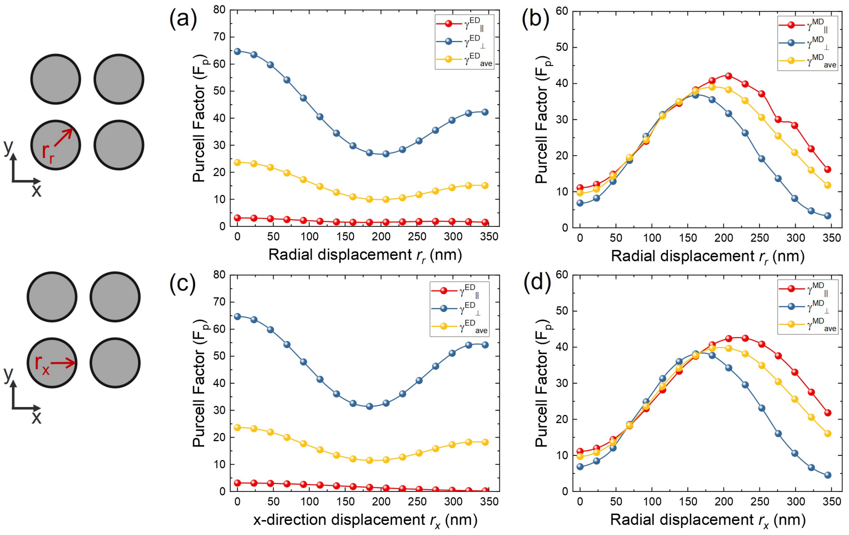

Figure S2: FEM simulated Purcell factor at λ=1540 nm for electric and magnetic dipoles as a function of radial (a,b) and x-direction (c,d) displacement inside the SiO_2 nanoslot for a square array of 49 nanopillars with r=390 nm and a_0=1000 nm. The red and blue dots indicate dipoles with parallel ($\gamma_{||}$) and perpendicular ($\gamma_{\perp}$) orientation with respect to the Si interface, respectively. The yellow dots indicate dipoles with averaged orientation.

The variation of the Purcell factor with respect to the electric and magnetic dipole position inside the silica nanoslot is shown in Figure S2. Although both electric and magnetic emitters exhibit a modulation of the simulated radiative decay rate as a function of the radial distance from the center of the nanopillar, ED and MD variations are in phase opposition. As a consequence the Purcell factor enhancement tends to become uniform for an emitter like Er^{3+} with mixed ED and MD contributions to the radiative transition at λ=1540 nm. For example, the Purcell factor for a square array of 49 nanopillars with r=390 nm and a_0=1000 nm, obtained averaging all the emitter positions in the nanoslot ($F_{p,ave}$=10.1), is almost equal to the one obtained with a single dipole at the center of the nanoslot ($F_{p,cent}$=10.2). Therefore a dipole in the center of the nanoslot with averaged orientation can represent a good approximation for the description of the radiative decay rate variation in a SiO_2 nanoslot homogeneously doped with Er^{3+} ions.

Finite-size effect: number of nanopillars in the simulated domain

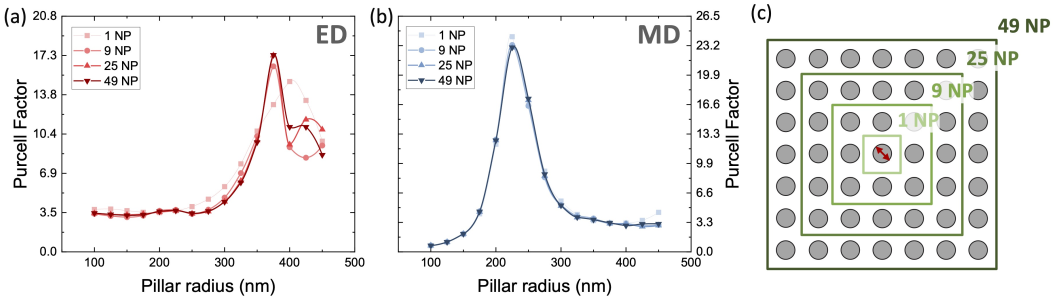

Figure S3: Finite-size effect on the simulated Purcell factor for an electric (a) and a magnetic (b) dipole with averaged orientation for 1, 9, 25, and 49 slotted silicon nanopillars in the simulation domain. The nanopillars are arranged in a finite square array with a_0=1000 nm. (c) A schematic representation of the configurations with 1, 9, 25, 49 nanopillars in the simulated domain. The red arrow indicates the slotted nanopillar with the emitter at the centre of the square array.

To evaluate the influence of the neighboring nanopillars on the decay rate modification (finite-size effect), a set of non periodic configurations with a growing number of nanopillars arranged in a square array with a_0=1000 nm around the one with the emitter have been simulated. Figures S3(a) and (b) report the Purcell factor for the 4 simulated configurations (i.e., 1, 9, 25, and 49 nanopillars in the domain) for electric and magnetic dipoles with averaged orientation at the center of the SiO_2 nanoslot. The simulated Purcell factor results almost unaffected by the presence of the first, second, and third nearest neighbours in the simulation domain (a small variation can be observed for the electric dipole for $r>350$ nm), indicating that the periodic lattice has a small influence on the slotted nanopillar Purcell factor.

Er^{3+} PL intensity and lifetime vs. angular and wavelength resolution

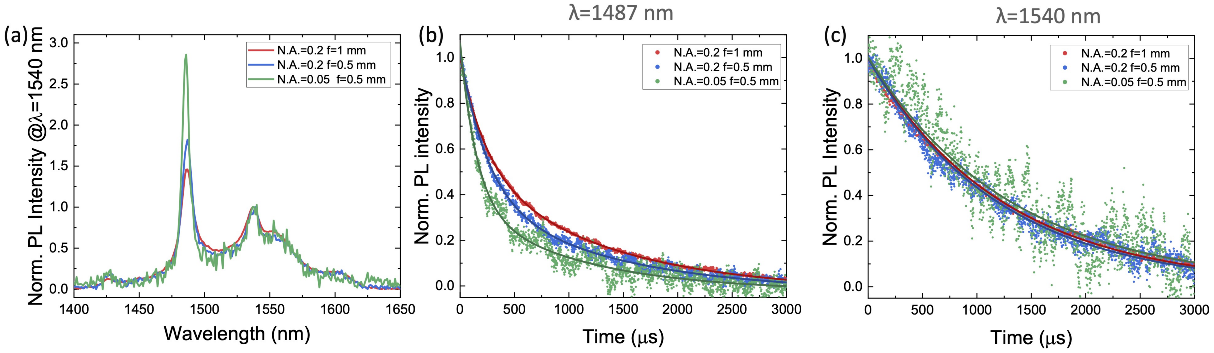

Figure S4: Influence of the numerical aperture of the collection lens and of the monochromator slits width on the PL spectrum (normalized at the value at λ=1540 nm) (a), the Er^{3+} lifetime at λ=1487 nm (b) and at λ=1540 nm (c) for the sample with r=360 nm and a_0=800 nm.

Figure S4(a) reports the evolution of the PL intensity spectrum (normalized at λ=1540 nm) for different numerical apertures of the collection lens and monochromator slits widths (f). Due to the high-Q factor of the mode at λ=1487 nm, the PL intensity enhancement is more prominent when the width of the monochromator slits and the NA of the collection lens is minimum. The PL temporal decay at λ=1487 nm and λ=1540 nm are shown in Figures S4(b) and (c), respectively. The experimental data were fitted with a double exponential function (i.e., $I_{PL}(t) = A_1 e^{t/\tau_1} + A_2 e^{t/\tau_2}$ where A_1+A_2=1) with τ_1=150±10 μs and τ_2=1.40±0.05 ms. The amplitude of the τ_1=150 μs component (A_1) at λ=1487 nm increases upon the decrease of the collection angular acceptance and the monochromator slits width, varying from A_1=0.62±0.02 for the lowest set-up resolution (red dots) to A_1=0.90±0.02 when f=0.5 mm and NA=0.05 (green dots). The decay rate measured at λ=1540 nm remains constant for all the explored experimental parameters, with a negligible amplitude of the τ_1=150 μs component.

Field intensity enhancement maps in the SiO_2 slot

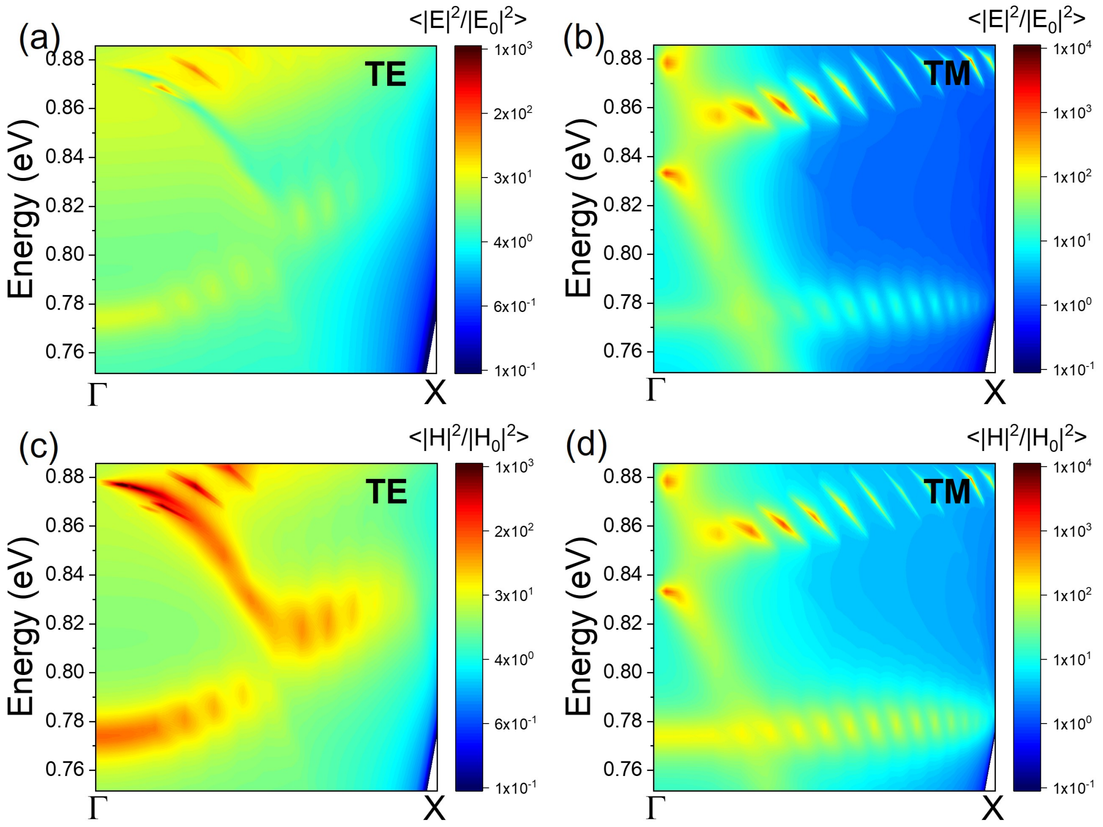

Figure S5: Simulated averaged electric ((a) and (b) panels) and magnetic ((c) and (d) panels) field intensity enhancement in the SiO_2 nanoslot for a TE- and TM-polarized plane wave impinging on the nanopillar array along the Γ-X directions in the reciprocal lattice, for the sample with r=360 nm and a_0= 800 nm.

Field intensity enhancement and reflectance at λ=1487 nm

Figure S6(a) reports the FEM simulated electric field enhancement as a function of the incident radiation energy for a TM-polarized plane wave impinging at θ=0.1° on the nanopillar array with r=360 nm and a_0=800 nm. A field intensity enhancement $< |\mathbf{E}|^2/|\mathbf{E}_0|^2 >$ higher than 10^5 has been computed at the resonance energy E_{res}=0.8364 eV (i.e., λ_{res}=1487 nm). The width w=4.17x10^{-6} eV (corresponding to $\Delta\lambda \sim 0.008$ nm) and the Q-factor Q=2x10^5 of the resonance have been calculated by the Lorentzian fit. The reflectance of the array

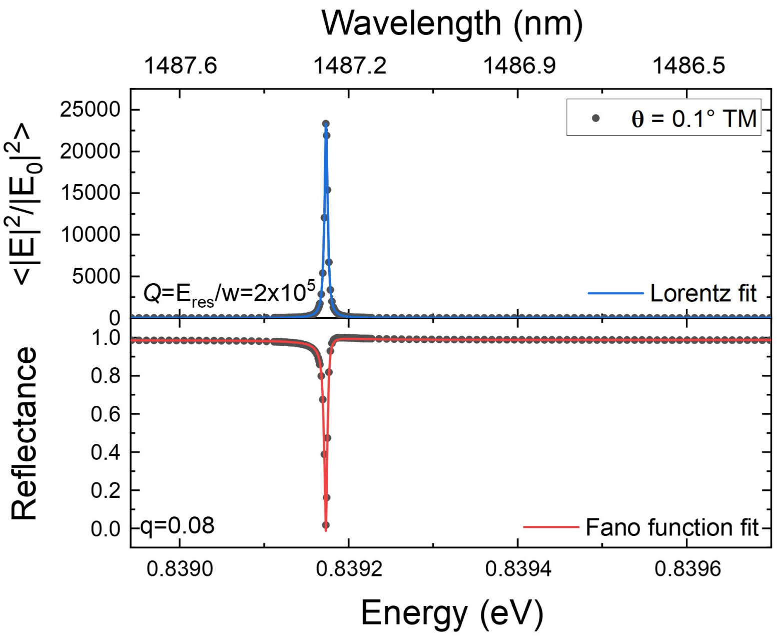

Figure S6: Volume average of the normalized electric field intensity enhancement in the SiO_2 slot (upper panel) and far-field reflectance (lower panel) for a TM-polarized plane wave impinging at θ=0.1° on the square array of slotted silicon nanopillars with r=360 nm, a_0=800 nm. The blue line in the upper panel indicates the fit of the resonance with a Lorentzian function, while the red line in the lower panel indicates the Fano function fit of the reflectance.

of nanopillars is shown in Figure S6(b). The reflectance spectrum exhibits a Fano-like line-shape typical of optical quasi-BIC resonances.[2] The simulated data were fitted with a Fano function.

Influence of the lattice parameter on the Q-factor

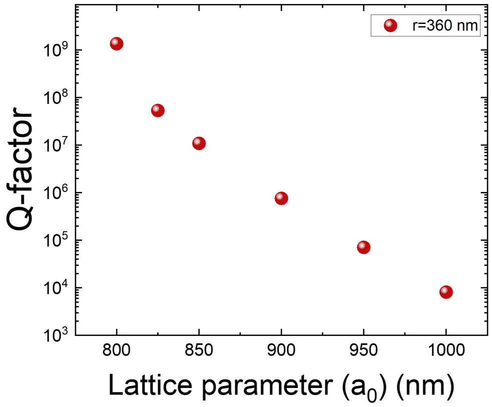

Figure S7: Quasi-BIC Q-factor as a function of the lattice parameter (a_0) for the sample with r=360 nm.

Near-field maps at λ=1487 nm

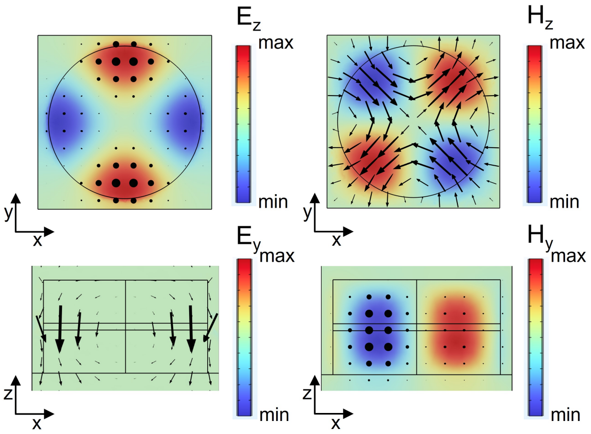

Figure S8: z- (upper panels) and y-components (lower panels) of the electric (left panels) and magnetic fields (right panels) at λ=1487 nm for the sample with r=360 nm and a_0=800 nm. Black arrows indicate the direction of the electric and magnetic fields.

Field distribution in the nanopillar with and without the slot

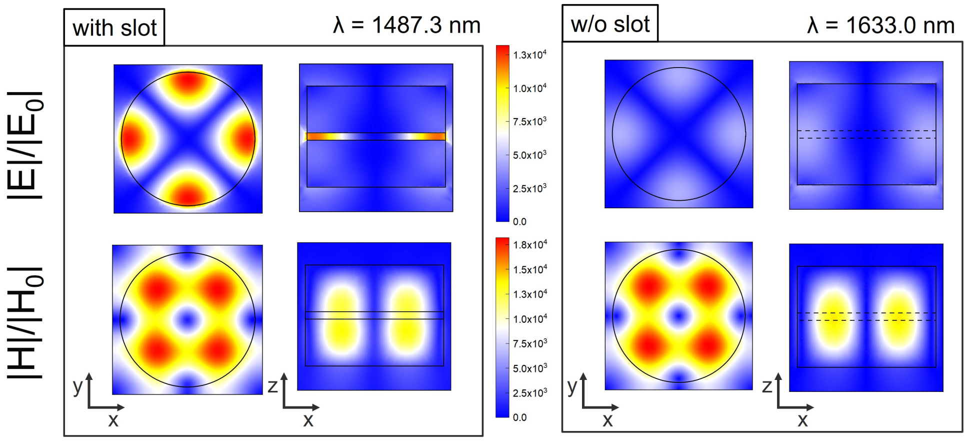

Figure S9: Calculated electric and magnetic field enhancement in the silicon nanopillar with (left panel) and without (right panel) the SiO_2 slot at λ=1487.3 nm and λ=1633.0 nm, respectively.

Electric and magnetic local fields were calculated by FEM simulations for a plane wave impinging on nanopillar arrays with (left panel) and without (right panel) the SiO_2 slot, from the air half-space, at normal incidence, in correspondence to the BIC resonance wavelengths. The nanopillar without the SiO_2 slot (r=360 nm and h=430 nm) supports the quadrupole BIC resonance at a slightly longer wavelength (λ=1633 nm) than the one with the SiO_2 slot, due to the refractive index variation from n_{SiO_2}=1.44 to n_{Si}=3.1. The magnetic field enhancement has an almost identical field distribution for both the nanostructures with a maximum value of $|H|/|H_0|\sim10^4$. On the contrary, the spatial distribution of the electric field enhancement clearly shows that the low index slot strongly enhances the field in the slot, that is where the Er emitters are placed with an electric field amplification that reaches $|E|/E_0|=1.2\times10^4$, i.e., a value $\sim$3 times higher with respect to nanostructure without the slot.

Effect of nanofabrication imperfections on the Q-factor

The theoretical Q-factor of quasi-BIC modes is experimentally reduced by two main limitations: (i) nanofabrication imperfections and (ii) the instrumental resolution (i.e., the

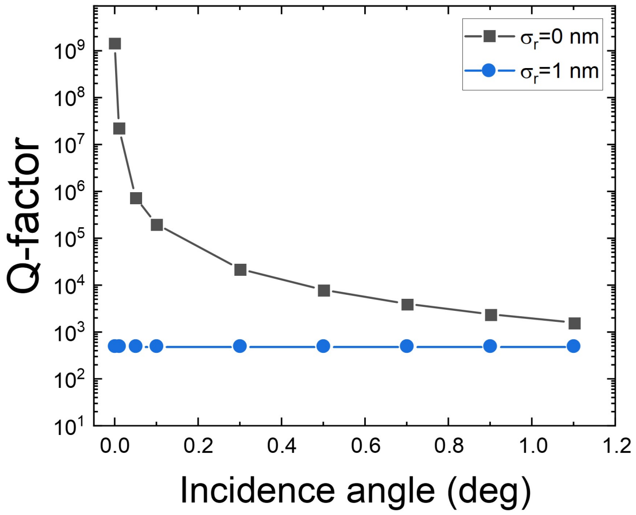

Figure S10: The effect of radii distribution on the simulated Q-factors as a function of the incident angle for a TM-polarized plane wave impinging on the nanopillar array with r=360 nm and a_0=800 nm.

finite angular and spectral resolution of the detection set-up). To compare theory with experiments, it is necessary to consider both the peak value of the Q-factor and its angular dependence (i.e., the integrated value in the collection angle). One way of modelling the fabrication-induced effects on the optical response of the nanoantennas array is to introduce random geometrical variations for each unit cell independently. However, this approach would lead to a prohibitively large simulation domain. Instead, we evaluated the Q-factor and resonance wavelength variations due to small modifications of the nanostructure geometry and used convolution to predict the final optical response of the nanoantennas array.

At first, we evaluated the influence of a distribution of nanopillar radii. To do so, we consider the sample with r=360 nm and a_0=800 nm, and assumed a standard deviation of σ_r=1 nm for the nanopillar radius. Then, FEM simulations were performed to evaluate the Q-factor and the resonance wavelength shift due to the variation of 1 nm in the nanopillar radius for the BIC mode with Q$\sim 10^9$. While the Q-factor remains almost unaltered, the resonance wavelength shifts of about λ=3 nm for a plane wave at normal incidence. It is worth noting that the resonance is more stable with respect to variations in the nanopillar height h_{tot} and the lattice parameter a_0. For Δh_{tot}=1 nm and Δa_0=1 nm, the Q-factor

remains unaltered, while the resonance wavelength shifts of $\Delta\lambda$=0.7 nm and $\Delta\lambda$=0.02 nm, respectively. The convolution of the Lorentzian quasi-BIC resonance with the wavelength shift due to the Gaussian dispersion of nanopillar radii, can be used as an estimate of the decrease of the Q-factor due to nanofabrication imperfections. Figure S10 reports the effect of the radii dispersion on the simulated Q-factors as a function of the incident angle for a TM-polarized plane wave impinging on the nanopillar array with r=360±1 nm and a_0=800 nm. Nevertheless, it is important to stress that this estimate can be assumed as an upper bound for the decrease of the Q-factor since it was calculated by simulating a set of arrays with perfectly identical nanoparticles with different radii rather than an array with a distribution of nanopillar radii, which cannot be simulated by applying periodic boundary conditions to the simulation domain. Moreover, even if the peak value of the Q-factor at θ=0° decreases significantly, the angularly integrated value (more correlated with the experimental data) remains more stable and keeps the measured Q-factor high. Furthermore, the arrays under investigation in the present work are 400x400 μm large, therefore, the finite size effect of the periodic structure plays a marginal role in the Q-factor of the quasi-BIC modes.[3]

Er^{3+} ED and MD PL intensity spectra

Due to the strongly mixed ED and MD radiative emission of Er^{3+} in the NIR, an additional set of samples have been nanofabricated to calculate the Er^{3+} PL emission spectra for the limiting cases of η_{ED}=1 and η_{MD}=1. To this purpose, a 400 nm thick SiO_2 layer was deposited by magnetron sputtering on top of an optically thick Au layer (t_{Au}=200 nm). A thin Er-doped SiO_2 layer ($t_{Er:SiO_2}$=20 nm) has been placed at three different distances (z_0) from the metal film. A schematic representation of the sample structure is shown in Figure S11(a).

The presence of an interface in close proximity to the emitter will influence both the electric and the magnetic LDOS, and therefore the ED or MD decay rates can be unbalanced controlling the emitter distance from the interface. Figure S11(b) reports the electric and

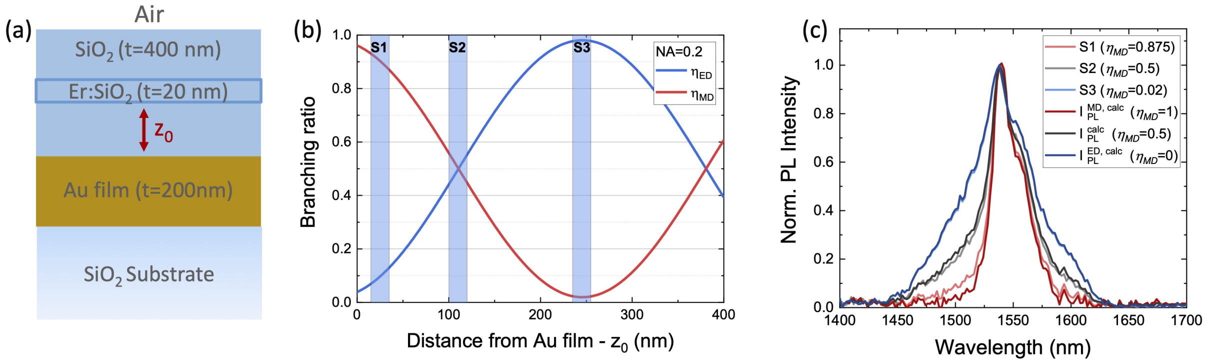

Figure S11: Er^{3+} emission near a gold interface. (a) Sketch of the samples geometry. (b) ED and MD branching ratios for an isotropic emitter computed with the CDO analytical model for a collection set-up with numerical aperture NA=0.2. (c) Measured and calculated Er^{3+} emission spectra in front of a gold mirror as a function of the branching ratio.

magnetic dipole branching ratio for an isotropic emitter with λ_{em}=1540 nm in front of a gold mirror calculated by the CDO analytical model[4] for a collection set-up with NA=0.2. Three Er:SiO_2-Au distances were selected corresponding to η_{MD}=0.875, η_{MD}=0.5, and η_{MD}=0.02 (samples labelled S1, S2, and S3, respectively). The experimentally measured Er^{3+} emission spectra of the three samples with different magnetic branching ratios are reported in Figure S11(c). Despite the homogeneous broadening due to the room temperature emission, the line-shapes of the three spectra clearly differ depending on the Er^{3+} branching ratio. Hence, assuming that the measured spectrum is a linear combination of the limiting cases with η_{ED}=1 and η_{MD}=1,[5] i.e., $I_{PL}^{exp}(\lambda) = (1-\eta_{MD})I_{PL}^{ED}(\lambda) + (\eta_{MD})I_{PL}^{MD}(\lambda)$, with η_{MD}=1-η_{ED}, $I_{PL}^{ED}(\lambda)$ and $I_{PL}^{MD}(\lambda)$ can be calculated from the two corresponding measured spectra (blue and red lines in Figure S11(c)). It is worth noticing the excellent agreement between the measured and calculated spectra with η_{MD}=0.5 (light vs. dark black lines in Figure S11(c)), which is a further cross-check of the $I^{ED}(\lambda)$ and $I^{MD}(\lambda)$ deconvolution procedure.

References

(1) Palik, E. D. *Handbook of optical constants of solids*; Academic press, 1998; Vol. 3.

(2) Bogdanov, A. A.; Koshelev, K. L.; Kapitanova, P. V.; Rybin, M. V.; Gladyshev, S. A.;

Sadrieva, Z. F.; Samusev, K. B.; Kivshar, Y. S.; Limonov, M. F. Bound states in the continuum and Fano resonances in the strong mode coupling regime. *Advanced Photonics* **2019**, *1*, 1.

(3) Liu, Z.; Xu, Y.; Lin, Y.; Xiang, J.; Feng, T.; Cao, Q.; Li, J.; Lan, S.; Liu, J. High-Q quasibound states in the continuum for nonlinear metasurfaces. *Physical review letters* **2019**, *123*, 253901.

(4) Chance, R.; Prock, A.; Silbey, R. Molecular fluorescence and energy transfer near interfaces. *Adv. Chem. Phys* **1978**, *37*, 65.

(5) Choi, B.; Iwanaga, M.; Sugimoto, Y.; Sakoda, K.; Miyazaki, H. T. Selective plasmonic enhancement of electric-and magnetic-dipole radiations of Er ions. *Nano Letters* **2016**, *16*, 5191–5196.
